# Identification and functional analysis of *Dmrt1* gene and the *SoxE* gene in the sexual development of sea cucumber, *Apostichopus japonicus*

**DOI:** 10.3389/fgene.2023.1097825

**Published:** 2023-01-19

**Authors:** Bing-Zheng Liu, Jing-Jing Cong, Wei-Yi Su, Zhen-Lin Hao, Zhi-Hui Sun, Ya-Qing Chang

**Affiliations:** Key Laboratory of Mariculture and Stock Enhancement in North China’s Sea, Ministry of Agriculture and Rural Affairs, Dalian Ocean University, Dalian, Liaoning, China

**Keywords:** RNAi, sea cucumber, gonad development, DMRT, SoxE

## Abstract

Members of the Doublesex and Mab-3-related transcription factor (*Dmrt*) gene family handle various vital functions in several biological processes, including sex determination/differentiation and gonad development. *Dmrt1* and *Sox9* (*SoxE* in invertebrates) exhibit a very conserved interaction function during testis formation in vertebrates. However, the dynamic expression pattern and functional roles of the *Dmrt* gene family and *SoxE* have not yet been identified in any echinoderm species. Herein, five members of the *Dmrt* gene family (*Dmrt1*, *2*, *3a*, *3b* and *5*) and the ancestor *SoxE* gene were identified from the genome of *Apostichopus japonicus*. Expression studies of *Dmrt* family genes and *SoxE* in different tissues of adult males and females revealed different expression patterns of each gene. Transcription of *Dmrt2*, *Dmrt3a* and *Dmrt3b* was higher expressed in the tube feet and coelomocytes instead of in gonadal tissues. The expression of *Dmrt1* was found to be sustained throughout spermatogenesis. Knocking-down of *Dmrt1* by means of RNA interference (RNAi) led to the downregulation of *SoxE* and upregulation of the ovarian regulator *foxl2* in the testes. This indicates that *Dmrt1* may be a positive regulator of *SoxE* and may play a role in the development of the testes in the sea cucumber. The expression level of *SoxE* was higher in the ovaries than in the testes, and knocking down of *SoxE* by RNAi reduced *SoxE* and *Dmrt1* expression but conversely increased the expression of *foxl2* in the testes. In summary, this study indicates that *Dmrt1* and *SoxE* are indispensable for testicular differentiation, and *SoxE* might play a functional role during ovary differentiation in the sea cucumber.

## 1 Introduction

The sea cucumber *A. japonicus* is an important nutritional seafood and is regarded as a precious traditional Chinese medicine (Yan J. et al., 2013; Yu Z. et al., 2014; Yang et al., 2015). The sea cucumber is widely distributed in the coastal regions of China, Far Eastern Russia, Japan, and Korea (Tian et al., 2017; Chen and Chang, 2015; Yan J. et al., 2013; Yu Z. et al., 2014), and its scale of breeding has continued to expand. Compared with the female sea cucumber, the male might have an advantage in terms of immunocompetence (Jiang et al., 2017; Jiang et al., 2019). Recently, several genes related to gonadal development have been identified, such as *piwi* (Sun et al., 2021), *vasa* (Yan M. et al., 2013) and *foxl2* (Sun et al., 2022), and a hypothetical XX/XY sex determination system has been proposed (Wei et al., 2021). However, the mechanisms underlying sex determination and sex differentiation in the sea cucumber remain a mystery.

Identification of sex-related genes is an effective method to reveal the genetic basis of sex determination/differentiation in the sea cucumber. The *Dmrt* gene family encodes a large family of crucial transcription factors containing one or several conserved DM domains; this family was first identified in fruit fly (*Drosophila melanogaster*) (*Arthropoda*) (Burtis and Baker, 1989). The *Dmrt* gene family is involved in various biological processes, including sex determination, sex differentiation, testicular development, and embryo development (Dong et al., 2020). To date, the *Dmrt* gene family has been identified in diverse organisms and members of the *Dmrt* gene family exhibit substantial variation in different organisms. For instance, eight *Dmrt* genes (*Dmrt1*, *Dmrt2*, *Dmrt3*, *Dmrt4*, *Dmrt5*, *Dmrt6*, *Dmrt7* and *Dmrt8*) have been identified in humans (*Homo sapiens*) (Ottolenghi et al., 2002) and mice (*Mus musculus*) (Kim et al., 2003). Six *Dmrt* genes, including *Dmrt1*, *Dmrt2*, *Dmrt3*, *Dmrt4*, *Dmrt5* and *Dmrt6,* have been identified in tuatara (*Sphenodon punctatus*), and *Dmrt1* is regarded as a crucial sex determination/differentiation gene in *S. punctatus* (Wang et al., 2006). Five *Dmrt* genes (*Dmrt1*, *Dmrt2*, *Dmrt3*, *Dmrt5* and *Dmrt6*) have been identified in fish (Dong et al., 2020). In amphibians, five *Dmrt* genes (*Dmrt1*, *Dmrt2*, *Dmrt3*, *Dmrt4* and *Dmrt5*) have been identified (Bewick et al., 2011; Watanabe et al., 2017). Due to teleost-specific whole-genome duplication (TGD) and rapid gene loss following TGD during evolution, the number of *Dmrt* genes is species-specific in teleost; there are five *Dmrt* genes in puffer fish (*Takifugu rubripes*) and zebrafish (*Danio rerio*) (Yamaguchi et al., 2006) but six *Dmrt* genes in Asian sea bass (*Lates calcarifer*), spotted gar (*Lepisosteus oculatus*) and channel catfish (*Ictalurus punctatus*) (Dong et al., 2020). Multiple *Dmrt* genes have also been identified from diverse invertebrate organisms. Seven *Dmrt* genes were identified both in Chinese mitten crab (*Eriocheir sinensis*) (*Arthropoda*), freshwater prawn (*Macrobrachium rosenbergii*) (*Arthropoda*) and mud crab (*Scylla paramamosain*) (*Arthropoda*) by screening transcriptome data using the bioinformatics method (Abayed et al., 2019; Du et al., 2019; Wan et al., 2021).

In addition to the identification of *Dmrt* gene families, several studies have focused on cloning, expression analysis and functional study of single *Dmrt* gene, especially the *Dmrt1* gene. *Dmrt1* is regarded as a conserved male-specific gene and plays a critical role in sex determination and sex differentiation in various species (Kopp, 2012). *Sox9* (SRY-related HMG box gene 9) is a member of the *SoxE* subfamily and is another essential regulator of testis determination in many organisms (Hui et al., 2021). Both *Dmrt1* and *Sox9* lay downstream of the male sex differentiation pathway, and the expression of *Dmrt1* was found to be upregulated before *Sox9* during testis development (Elzaiat et al., 2014). *Dmrt1* positively regulates the transcription of the *Sox9b* gene by directly binding to a specific cis-regulatory element (CRE) (Wei et al., 2019; Vining et al., 2021). Knockdown of the male sex-determining gene *Dmrt1* leads to decreased *Sox9* expression, while overexpression of *Dmrt1* results in upregulation of *Sox9* expression in red-eared slider turtle (*Trachemys scripta*) (Ge et al., 2017). In vertebrates, at least three members, including *Sox8*, *Sox9* and *Sox10,* have been identified in the *SoxE* subfamily, but only one ancestral *SoxE* gene has been found in all studied invertebrate species (Heenan et al., 2016). The role of *SoxE* in the specification of the neural crest and the regulation of chondrogenesis has been revealed in invertebrates (McCauley, 2008); however, little is known about its function in reproduction and the interaction between *Dmrt1* and *SoxE*.

In the current study, five members of the *Dmrt* gene family and a single *SoxE* gene were identified, and their molecular characteristics and phylogenetics were systemically analysed. Moreover, their dynamic expression patterns in different adult tissues and during different gonadal developmental stages were analysed using real-time quantitative PCR (RT-qPCR). Finally, the function and underlying interaction between *Dmrt1* and *SoxE* in the process of gonadal development were explored using RNA interference (RNAi). These findings provide valuable information for understanding the sex determination and differentiation mechanisms of Echinodermata.

## 2 Materials and methods

### 2.1 Identification of *Dmr*t gene family members and the *SoxE* gene

The published genome (taxid: 307972) data of the sea cucumber at the scaffold level (Zhang et al., 2017) was downloaded from the NCBI database (https://www.ncbi.nlm.nih.gov/). A BLASTP search was then performed using the common conserved domain protein sequence of the DM domain (XM_030995783.1) and the High Mobility Group box (HMG box) (XP_786809.2) to screen the homologous genes of the *Dmrt* family and *SoxE*, respectively. The E-value was set to ≤ e− 5. The suspected members of the *Dmrt* gene family and *SoxE* gene were validated by blasting against the NCBI database, in order to assess the reliability of the analysis.

### 2.2 Sequence and phylogenetic analysis

The coding sequence of the *Dmrt* gene was predicted by ORF Finder online software (https://www.ncbi.nlm.nih.gov/orffinder)*.* BioEdit software was used to perform multiple sequence alignments and visual analysis of the *Dmrt* proteins in the sea cucumber. The sequences used in this study were downloaded from the NCBI database (Supplementary Table S1). Phylogenetic analysis was performed using MEGA-7 software with bootstrapping (1,000 replicates) and the neighbour-joining (NJ) method.

### 2.3 Real-time quantitative PCR (RT-qPCR)

Adult sea cucumbers were collected from the coastal areas of Dalian, China. No endangered or protected species were involved in this study. The expression patterns of the *Dmrt* genes and *SoxE* gene in different tissues were analysed by RT-qPCR. Briefly, total RNA from different tissues, including the tube feet, testes, intestines, ovaries, stomach, longitudinal muscle, coelomocytes and respiratory tree, were extracted using the Silica Membrane-based Vacuum Pump (SV) Total RNA Isolation System (Promega Z3100). The middle part of the intestine was used for gene expression analysis. According to previously reports (Sun et al., 2021), the gonads of *A. japonicus* are classified into four stages, including the early growing stage (Stage 1), growing stage (Stage 2), mature stage (Stage 3) and post-spawning stage (Stage 4). After gonadal histology analysis, gonadal tissues at different development stages were collected to examine the dynamic expression changes in *Dmrt1* and *SoxE*. Gonadal tissues at Stage 2 were used to detect tissue expression specificity.

RT-qPCR experiments were performed in 20-μl reactions containing 10 µL of Fast Start Essential DNA Green Master (Roche, Mannheim, Germany), 6.4 µL of sterile water, 0.8 µL of each 10 mM primer, and 2 µL of cDNA. The protocol was as follows: 95°C (10 min) for heat denaturing; then, 40 cycles of 95°C (15 s) and 60°C (1 min). The efficiency of primers discovery through standard curve formulation reached 98%. The housekeeping gene *NADH* was selected as the internal reference gene (Sun et al., 2021). Data were performed from three independent experiments. Each sample was analyzed in triplicates, and the relative expression levels of target genes were calculated with the 2^—ΔΔCt^ method. For statistical analysis, one-way ANOVA was calculated with SPSS software after the normal distribution and homogeneity of variance test (SPSS Inc.), and a probability (*p*) of ≤0.05 was considered statistically significant. The primers used in this study were designed using online software (http://biotools.nubic.northwestern.edu/OligoCalc.html) (Supplementary Table S2).

### 2.4 RNA interference

Gene-specific dsRNAs for the *Dmrt1* and *SoxE* genes were designed using an online platform (https://www.dkfz.de/signaling/e-rnai3/). DsRNAs were synthesised using a T7 RiboMAX™ Express RNAi System (Promega), according to the manufacturer’s protocol. Three different dsRNAs were designed for each gene to ensure interference efficiency. The genetic sex of each sea cucumber was determined according to a previous report (Wei et al., 2021). In briefly, the tube feet tissues were used to extract genomic DNA by alkaline lysis method, then male-specific primers were used to amplify the male specific DNA marker, one specific band was amplified in males, but not in females. For the *Dmrt1* RNAi experiment, 24 male sea cucumbers with an average weight of 100 ± 20 g were selected and were randomly divided into two groups: *Dmrt1*-knockdown (*n* = 12) and control (*n* = 12). For the *SoxE* RNAi experiment, 24 male sea cucumbers with an average weight of 60 ± 10 g were selected. The 24 males were divided into two groups: *SoxE*-knockdown (*n* = 12) and control group (*n* = 12). The injection experiments were performed as previously reported with slight modification (Sun et al., 2021). Specifically, before injecting, three gene-specific dsRNAs were mixed, then 100 μg of gene-specific dsRNA per 50 g body weight was injected into each sea cucumber in the knockdown group. Meanwhile, sea cucumbers in the control group were injected with GFP dsRNA at the same concentration. For the *Dmrt1* RNAi experiment, three sea cucumbers from each group were removed randomly on the third day, seventh day and 10th day following the first injection. The gonads were surgically sampled for future research. In addition, repeat injections were performed on the third, sixth and ninth days following the first injection. For the *SoxE* RNAi experiment, three sea cucumbers from each group were removed randomly on the third, 7th and 23rd day following the first injection. The gonads were surgically sampled for future research. In addition, repeat injections were performed every 3 days after the first injection.

### 2.5 Gonadal histology

Gonadal tissues were surgically removed and fixed in 4% paraformaldehyde (PFA) at 4°C overnight. Then, they were washed three times in phosphate-buffered solution (PBS) and balanced in 30% sucrose at room temperature for 2 h. Next, the treated tissues were embedded in optimal cutting temperature (O.C.T) compound and frozen sections (5 μm) were cut and stained with haematoxylin/eosin. Digital photos were acquired under a Leica DM4B microscope

## 3 Results

### 3.1 Identification and characterization of *Dmrt* and *SoxE* genes in *A. japonicus*

Genomic information for *A. japonicus* is available from NCBI (assembly ASM275485v1) (Zhang et al., 2017). To identify the *Dmrt* and *SoxE* genes in *A. japonicus*, the protein sequences of the conserved DM domain and HMG box of *S. purpuratus* (XM_030995783.1 and XP_786809.2) were used as the queries to perform a BLASTP search, respectively. Five predicted *Dmrt* genes (PIK43860.1, PIK34621.1, PIK44057.1, PIK33706.1 and PIK41536.1) were identified. Each *Dmrt* gene contained a complete coding DNA sequence (CDS) with lengths of 1,029, 651, 945, 507 and 849 bp, respectively. All identified *Dmrt* proteins had a DM domain (Supplementary Figure S1A), but had a different number of exons. PIK41536.1, PIK43860.1 and PIK34621.1 contained just a single intron, PIK44057.1 had two introns and PIK33706.1 had no introns (Supplementary Figure S1B). The identity between *Dmrt* cDNA sequences is about 32 %. Likewise, a predicted *SoxE* gene encoding a protein of 459 amino acids was identified *AjSoxE*. As expected, there was a 71-amino acid HMG box domain in the protein sequence (Supplementary Figure S1C).

**FIGURE 1:**
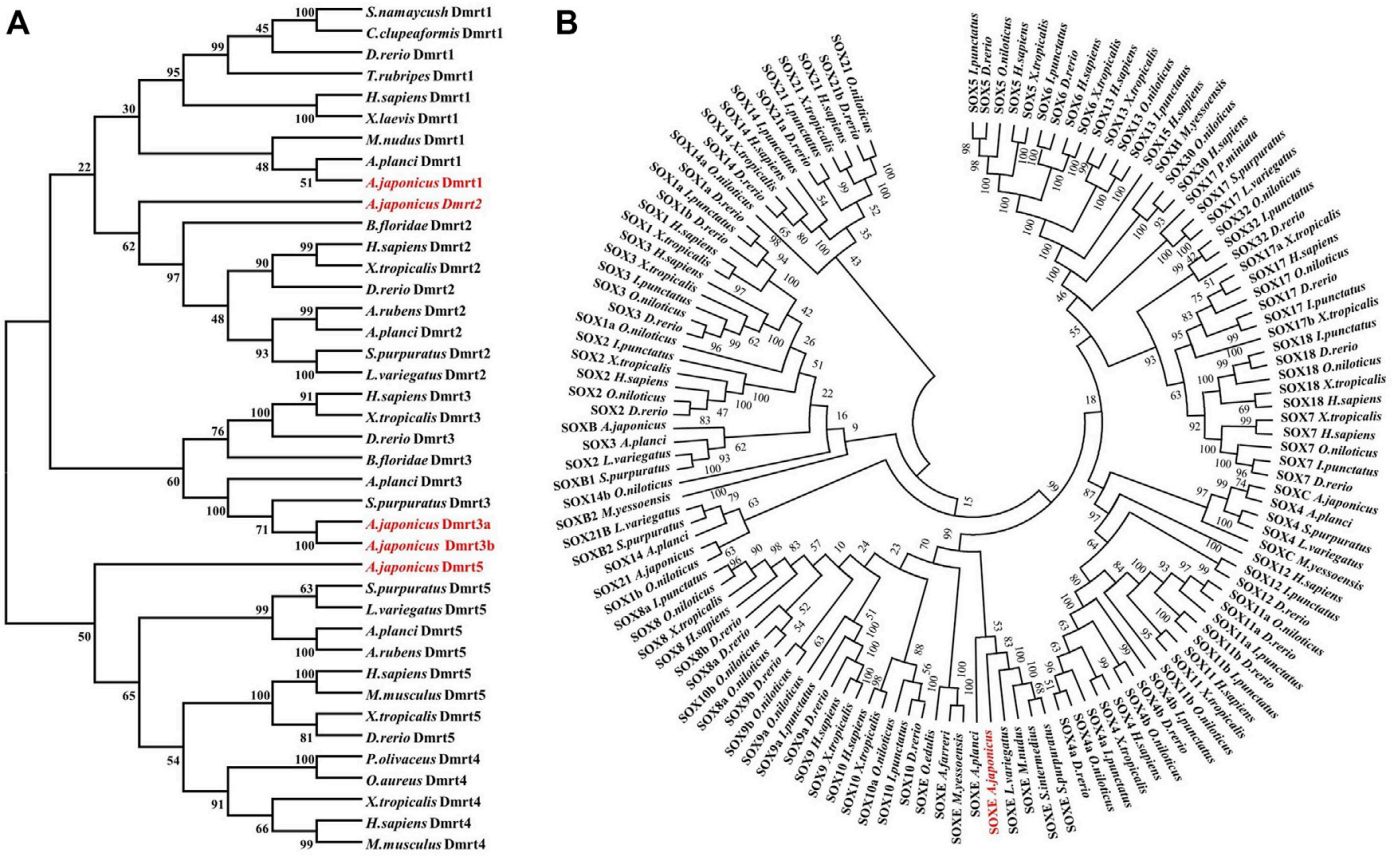
Phylogenetic analysis of the Dmrt and SOX protein family in *Apostichopus japonicus* **(A)** Dmrt.**(B)** SoxE. The phylogenetic tree was constructed with MEGA version 7.0 (1,000 replicates) by maximum likelihood analysis.

To define these five *Dmrt* genes, 40 *Dmrt* genes from 17 species were downloaded from the NCBI database and multiple protein sequence alignments and phylogenetic analyses were performed. The protein sequences of the *Dmrt* genes exhibiting low similarity between species (4.94%–57.01%). However, the protein sequence of DM domain was highly conserved such that the similarity with other species was generally more than 60% (Supplementary Figures S2–S5). The phylogenetic analyses indicated that the *Dmrt* family genes can be classified into five branches (*Dmrt*1, -2, -3, -4 and -5). PIK41536.1 and PIK43860.1 were clustered into the *Dmrt1* and *Dmrt2* branches, and were named *AjDmrt1* and *AjDmrt2*, respectively. PIK34621.1 and PIK44057.1 were both clustered with *Dmrt3* homologs from other species, so were named *AjDmrt3a* and *AjDmrt3b*, respectively. The deduced amino acid sequences of PIK33706.1 were clustered into the *Dmrt5* branch and were defined as *AjDmrt5* (Figure 1A). Surprisingly, none of the *AjDmrt* genes clustered with *Dmrt4* homologs from other species’ branches. The topology of clades of *Dmrt1*, *Dmrt2*, *Dmrt3* and *Dmrt5* were basically consistent with the known taxonomic relationships among these species. Moreover, the phylogenetic analyses showed that *AjSoxE* was firstly grouped with *SoxE* in invertebrate species, then clustered into *Sox8*, *Sox9* and *Sox10* branches in jawed vertebrates (Figure 1B). This phylogenetic tree further confirms that the ancestral *SoxE* gene is replicated in vertebrates and has produced at least three members (*Sox8*, *Sox9*, and *Sox10*).

### 3.2 Expression of *Dmrts* and *soxE* in adult tissues in *A. japonicus*

**FIGURE 2:**
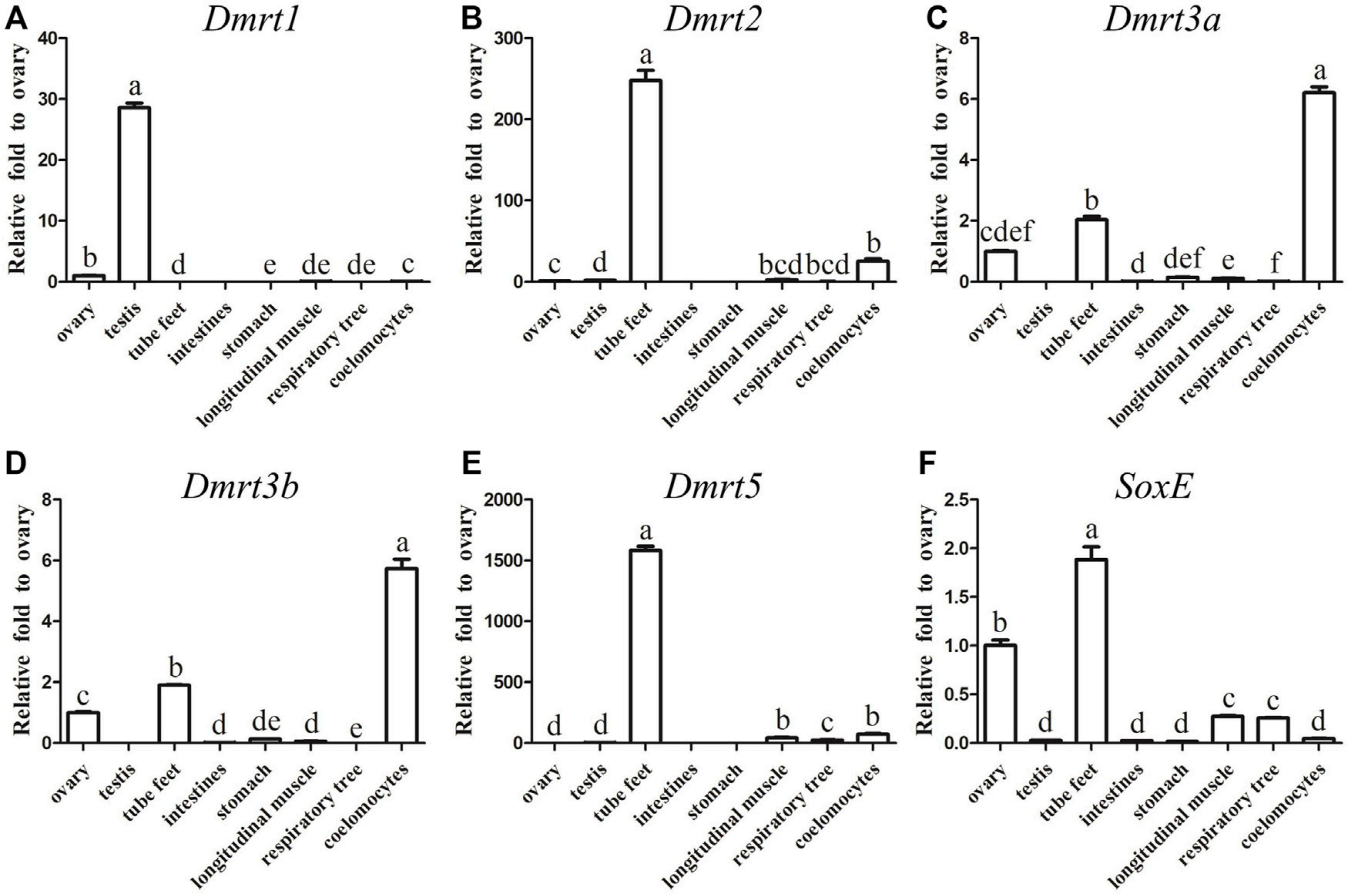
RT-qPCR analysis of *Dmrts* and *SoxE* Expression in adult tissues. NADH was used as internal control **(A)** Dmrt1. **(B)** Dmrt2. **(C)** Dmrt3a. **(D)** Dmrt3b. **(E)** Dmrt5. **(F)** SoxE. Data were performed from three independent experiments. Each bar represents mean ± SD. Different letters indicate significant differences between mean values (*p* ≤ 0.05), whilst shared letters indicate no significant difference.

In order to reveal the expression differences, the transcripts of all five *AjDmrt* genes and the *SoxE* gene in adult tissues were examined by RT-qPCR. Specific primers of five *AjDmrt* genes were designed in the parts with the greatest difference in the nucleic acid sequence. *AjDmrt1* was expressed predominantly in the testes, only low *AjDmrt1* expression was detected in other tissues, including the ovaries, stomach, tube feet, longitudinal muscle, respiratory tree, and coelomocytes (Figure 2A). In contrast, *AjDmrt2* was higher expressed in the tube feet (Figure 2B). *AjDmrt3a* and *AjDmrt3b* were expressed predominantly in the coelomocytes and tube feet, with low expression of *AjDmrt3a* and *AjDmrt3b* detected in other tissues, including the intestines, stomach, longitudinal muscle, ovaries, and respiratory tree (Figures 2C,D). The expression pattern of *AjDmrt5* in adult tissue was similar to that of *AjDmrt2*, with predominant expression in the tube feet and slight expression in the other analysed tissues (Figure 2E). Strikingly, the *SoxE* gene had the highest expression in the tube feet. Its expression differed significantly between the male and female gonads, such that a large number of transcripts were detected in the ovaries while *SoxE* was nearly undetectable in the testes (Figure 2F).

### 3.3 Expression profile of *Dmrt1* and *SoxE* during gonadal development in *A. japonicus*

**FIGURE 3:**
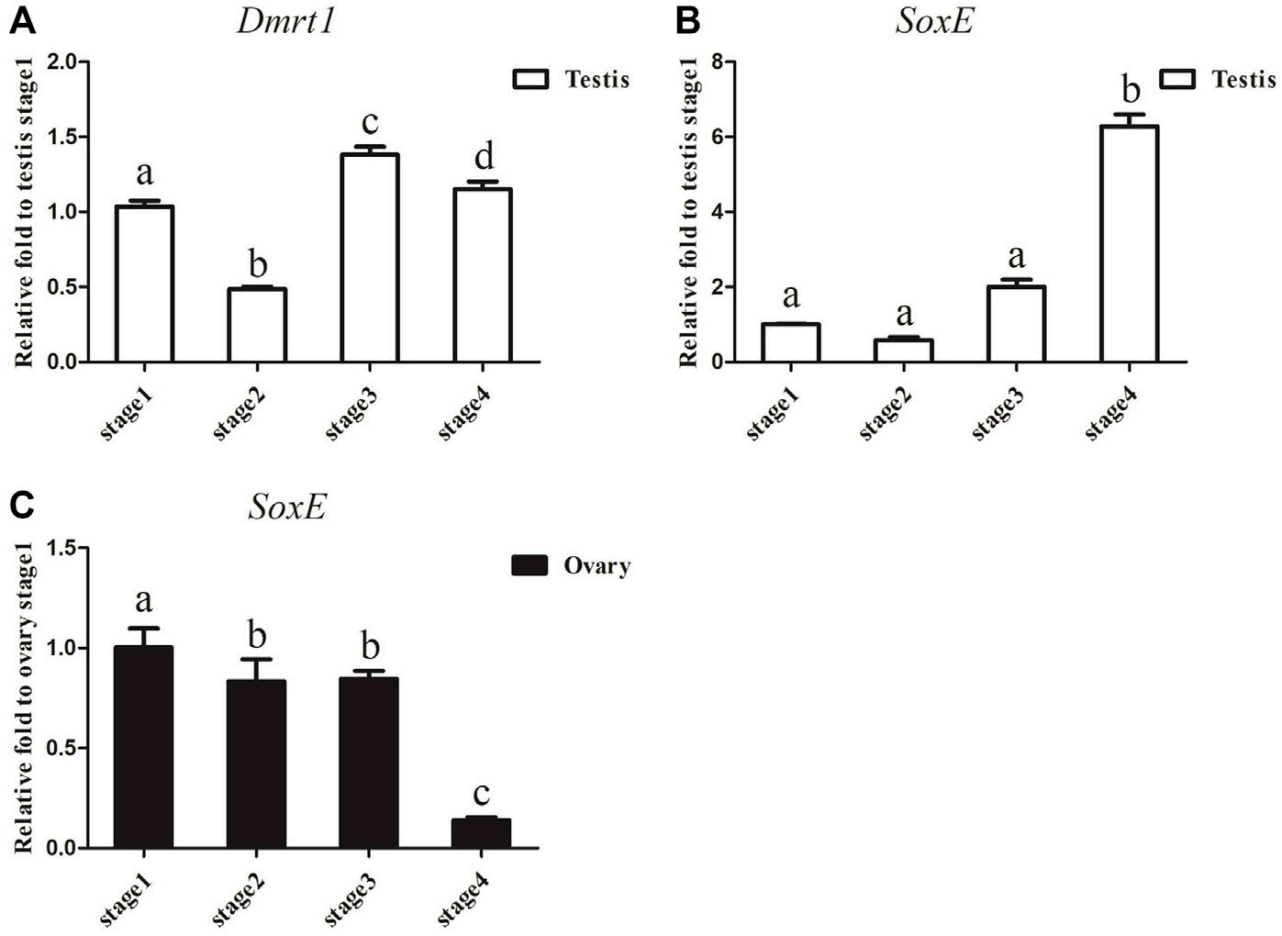
*Dmrt1* and *SoxE* expression in gonad. **(A)** RT-qPCR analysis of *Dmrt1* expression in testis at different development stages. **(B)** RT-qPCR analysis of *SoxE* expression in testis at different development stages. **(C)** RT-qPCR analysis of *SoxE* expression in ovary at different development stages. NADH was used as internal control. Data were performed from three independent experiments. Each bar represents mean ± SD. Different letters indicate significant differences between mean values (*p* ≤ 0.05), whilst shared letters indicate no significant difference.

Considering that the expression of *Dmrt1* and *SoxE* differed significantly between the ovaries and testes in *A. japonicus*, gonadal tissues at different development stages were collected to examine the dynamic expression changes in *Dmrt1* and *SoxE*. After gonadal histology analysis, gonadal tissues of four different development stages (i.e., the early growing stage (Stage 1), growing stage (Stage 2), mature stage (Stage 3) and post-spawning stage (Stage 4)) were obtained from male and female individuals, respectively. The expression of *Dmrt1* was continuously maintained at a high level during the spermatogenesis process and was significantly downregulated during Stage 2 in the testes (Figure 3A). *SoxE* expression in the testes at the corresponding stage was much lower than that of *Dmrt1*, and there were no significant differences between Stage 1, Stage 2 and Stage 3. Along with the occurrence of spermatogenesis, the expression level of *SoxE* increased up to 6.3-fold during Stage 4 against to Stage1 (Figure 3B). Meanwhile, a large amount of *SoxE* transcript was detected in Stage 1 ovary tissue, but the relative expression level continuously decreased together with the oogenesis development process, reaching the lowest in Stage 4 (Figure 3C).

### 3.4 Knockdown of *Dmrt1* by RNAi in male *A. japonicus*

**FIGURE 4:**
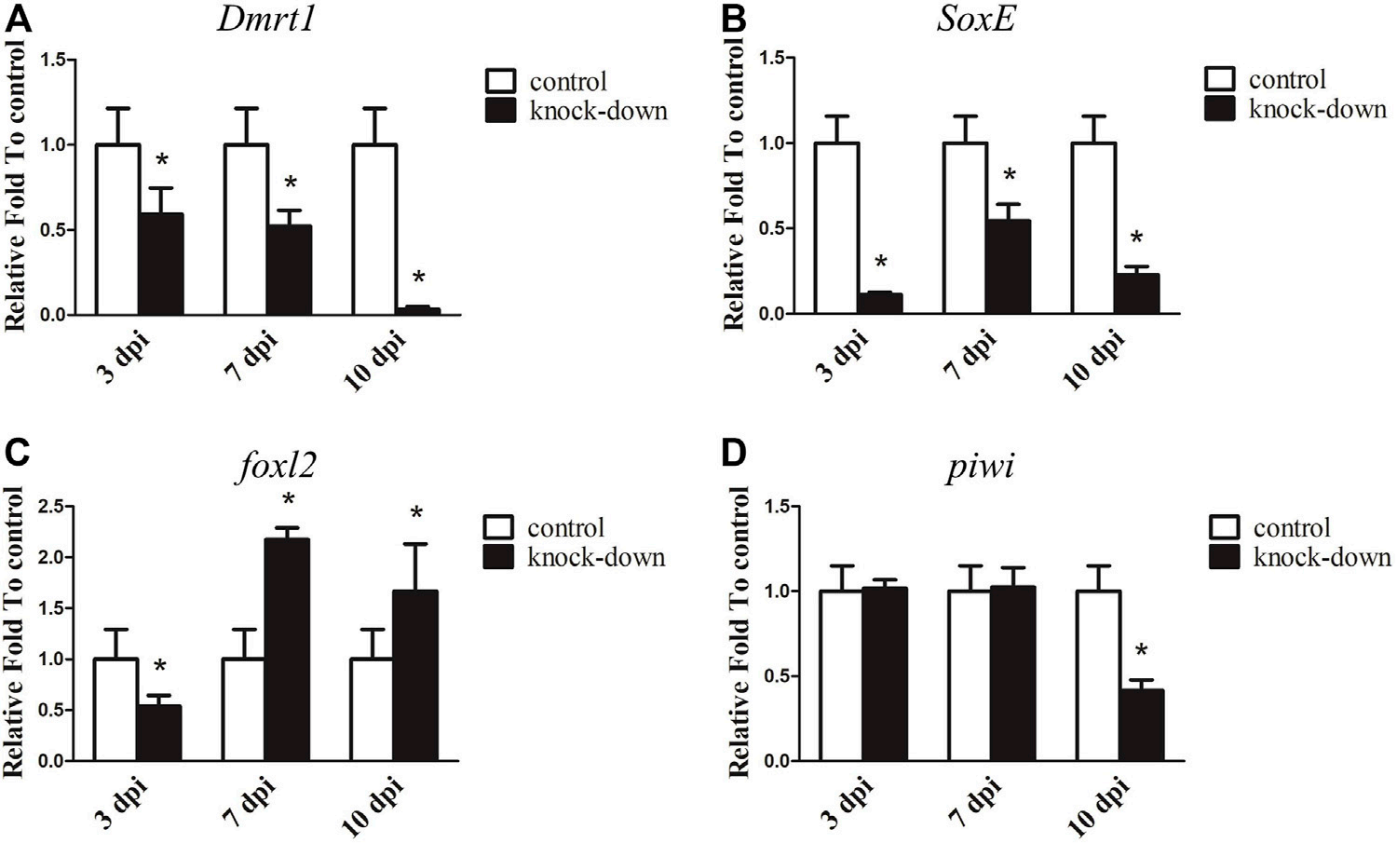
RNA interference (RNAi) of *Dmrt1* in testis. RT-qPCR detected mRNA levels of *Dmrt1* and other sex related genes after *Dmrt1* RNAi. **(A)** Dmrt1. **(B)** *SoxE*. **(C)** *foxl2.* **(D)** *piwi*. NADH was used as internal control. Data were performed from three independent experiments. Each bar represents mean ± standard deviation (SD). Asterisks (*) indicate significant differences (*p* ≤ 0.05) between knock-down and control. dpi: days post-injection.

RNAi was further utilised to examine the underlying function of *Dmrt1* in testes development in male *A. japonicus.* As shown in Figure 4A, the expression levels of *Dmrt1* significantly decreased, down 40.85% and 47.80% in the knockdown group compared to the control group at 3 days post-injection (dpi) and 7 dpi, respectively*.* On the 10 dpi, *Dmrt1* expression was only 3.31% of that in the control group. Compared to the control group, the transcripts of *SoxE* were reduced in the knockdown group by 88.65% at 3 dpi, 45.52% at 7 dpi and 77.10% at 10 dpi (Figure 4B). Moreover, the dynamic expression changes in *foxl2* and *piwi*, which have been reported to be involved in ovary differentiation and germ cell development, respectively (Ottolenghi et al., 2005; Gonzalez et al., 2021), were investigated. Interestingly, the expression level of *foxl2* was significantly downregulated (about 46.17%) in the knockdown group at 3 dpi, but sharply increased 1.67–2.17-fold at 7 dpi and 10 dpi (Figure 4C). The expression level of *piwi* did not significantly differ between the knockdown and control groups at 3 dpi and 7 dpi, but was significantly reduced by 58.36% at 10 dpi in knockdown adults (Figure 4D). Although the expression levels of genes involved in testis differentiation, ovary differentiation and germ cell development showed dynamic changes after knockdown of *Dmrt1* by RNAi, no obvious histological changes and no apoptosis cells were observed in the knockdown testes (Supplementary Figure S6).

### 3.5 knockdown of *SoxE* by RNAi in male *A. japonicus*

**FIGURE 5:**
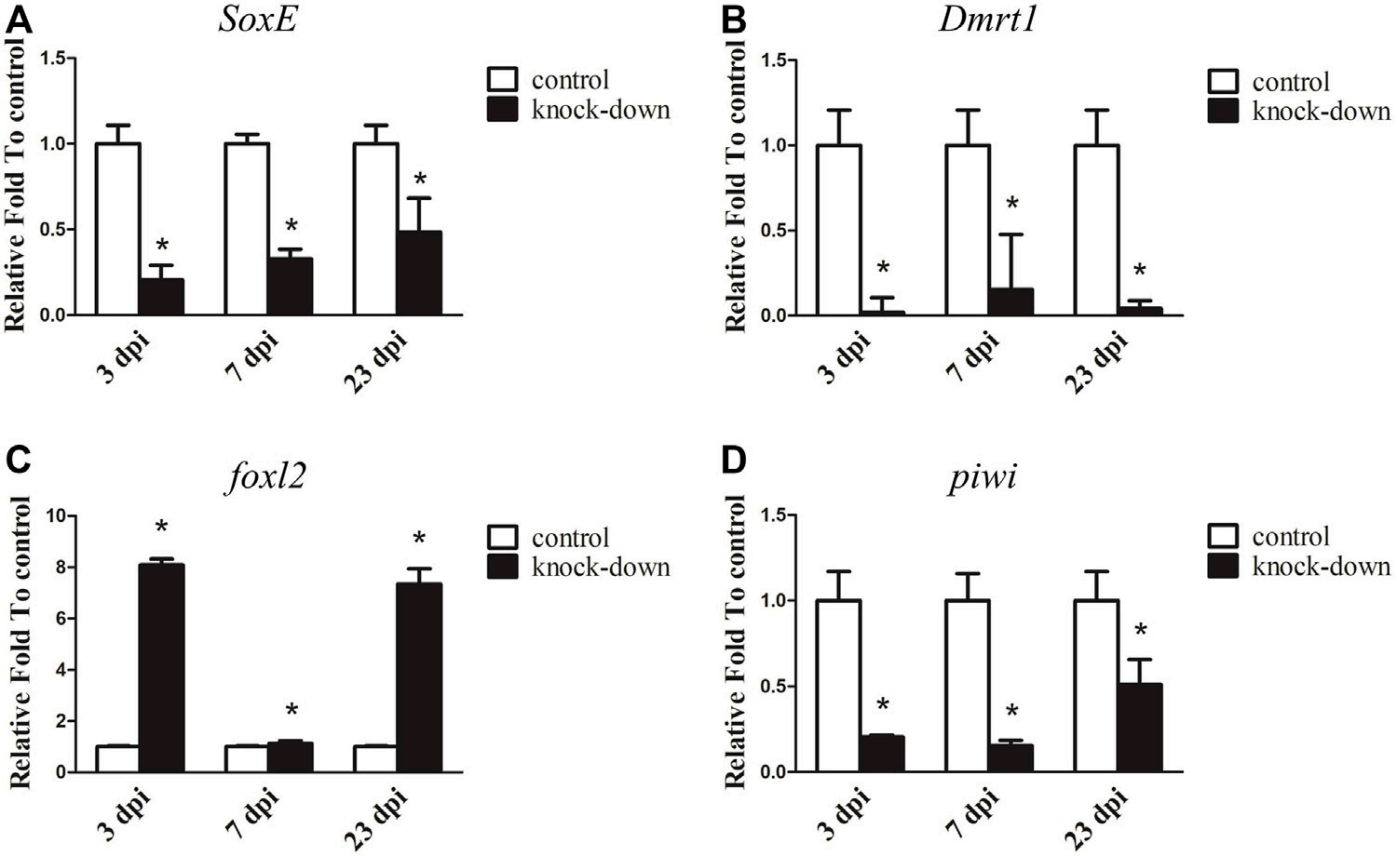
RNA interference (RNAi) of *SoxE* in testis. RT-qPCR detected mRNA levels of *SoxE* and other sex related genes after *SoxE* RNAi. **(A)** *SoxE*. **(B)** *Dmrt1*. **(C)** *foxl2.* **(D)** *piwi*. NADH was used as internal control. Data were performed from three independent experiments. Each bar represents mean ± standard deviation (SD). Asterisks (*) indicate significant differences (*p* ≤ 0.05) between knock-down and control. dpi: days post-injection.

Since *SoxE* has been reported to play a conserved role in sexual differentiation, and it shows a sexually dimorphic expression pattern in the gonads of *A. japonicus,* its function during testis development was investigated by RNAi in an adult male individual. As shown in Figure 5A, the transcript level of *SoxE* was significantly decreased by 79.46% with *SoxE* knockdown compared to the control at 3 dpi. Its expression was continuously downregulated at 7 dpi and 23 dpi. In addition, the expression of *Dmrt1* was sharply decreased to almost an undetectable level in the knockdown testes (Figure 5B). Conversely, the expression level of *foxl2* was increased about 8.09-fold and 7.34-fold in the knockdown sample compared to the control sample at 3 dpi and 23 dpi, respectively. However, the expression level of *foxl2* was not significantly different compared to the control at 7 dpi (Figure 5C). The transcripts of *piwi* were significantly decreased by 79.42%, 84.60% and 48.87% at 3 dpi, 7 dpi and 23 dpi, respectively (Figure 5D ). However, no obvious histological changes or apoptosis cells were observed in the knockdown testes (Supplementary Figure S7).

## 4 Discussion

With the development of sequencing technology, genome-wide investigations of gene families have become common, and several gene families, including the *Dmrt* gene family (Zafar et al., 2019), *Sox* gene family (Yang et al., 2020), *DEAD-box* helicase family (Xu et al., 2021), *NRAMP* gene family (Tian et al., 2021), *TRAF* gene family (Zhang et al., 2020), *TGF-beta* gene family (Cao et al., 2018) and *TLR* gene family (Habib et al., 2021) have been identified in various species. The origin of the *Dmrt* gene family can be traced back to *coelenterates* in the animal kingdom (Bellefroid et al., 2013; Wexler et al., 2014), and their composition, expression, and function show species-specificity. Here, *Dmrt3* genes were duplicated in the *A. japonicu*s genome and *Dmrt3a* and *Dmrt3b* were produced, whereas *Dmrt4* was lost. In other species, *Dmrt4* and *Dmrt5* were clustered into a major branch (Wang et al., 2012; Dong et al., 2020), indicating that these genes possibly originated from a common ancestor of *Dmrt*. This loss or duplicated gene phenomenon possibly resulted from independent gene duplication (loss) during the evolution of the sea cucumber. In most vertebrates, the *Dmrt1*–*Dmrt3*–*Dmrt2*(2a) gene cluster is highly conserved and plays a key role in sex differentiation (Johnsen and Andersen., 2012; Dong et al., 2020). Since the existing sea cucumber genome is not assembled to the chromosome level (Zhang et al., 2017; Li et al., 2018), we failed to identify the *Dmrt1*–*Dmrt3*–*Dmrt2*(2a) conserved gene cluster (Supplementary Figure S8). Hence, a chromosome-level assembly of the sea cucumber (*A. japonicu*s) should be sequenced to further elucidate the linkages among the conserved gene cluster and better understand the sex-determination mechanism.

Two different expression patterns of *Dmrt1* have been identified, including testes-specific expression and sexually dimorphic expression. In most cases, *Dmrt1* is specifically expressed in the testes and plays essential roles in male-sex determination and testicular differentiation. Knockdown or knockout of *Dmrt1* leads to male-to-female sex reversal (Paul-Prasanth et al., 2006; Luo et al., 2015; Chakraborty et al., 2016). However, there are some situations in which *Dmrt1* shows a sexually dimorphic expression pattern, with slight expression in the ovaries and abundant expression in the testes; for example, in red-eared slider turtle (*Trachemys scripta*) (Ge et al., 2017). In sea cucumber, *Dmrt1* shares a similar expression pattern with the red-eared slider turtle, with higher expression in the testes than in the ovaries. In addition, its expression was found to be highly expressed throughout spermatogenesis, but declined at the growing stage (Figure 3A). *Dmrt1* expression was decreased at spermiation stage in comparison with growing and maturing stages in *Oncorhynchus mykiss* and *Odontesthes bonariensis* (Marchand et al., 2000; Fernandino et al., 2006). These differences may be due to the different reproductive strategies and different testis structure between sea cucumber and fish, and should be investigated in the future. Curiously, there appears to be greater expression of *SoxE* in the ovaries than the testes at the same developmental stages. Both *Dmrt1* and *Sox9* are necessary for male sexual development in several species, knockdown (off) of *SoxE* (*Sox9*) and *Dmrt1* leads to the disappearance of the male marker, ectopic expression of the ovarian regulator and the formation of an ovary-like structure (Barrionuevo et al., 2006; Kim et al., 2007; Krentz et al., 2009; Cui et al., 2017). In Nile tilapia knockdown of *Dmrt1* led to the downregulation of *Sox9b,* and two potential cis-regulatory elements (CREs) for the *Dmrt1* transcription factor were identified in the promoter of *Sox9b* (Wei et al., 2019). Loss of *DMRT1* will inhibit *SOX9* expression and activates *FOXL2* transcription factor, even can reprogram granulosa cells into Sertoli cells in mice (Matson et al., 2011). In most species, sex is determined by male regulatory gene network in which *Sry* activates *Sox9* and a female network involving *WNT/b-catenin* signalling (Matson et al., 2011), and *Dmrt1* recruits *Sox9* to reprogram sexual cell fate(Lindeman et al., 2021). Although no obvious histological changes were observed in *SoxE* and *Dmrt1* knockdown testes in the current study, the expression level of the ovarian regulator *foxl2* was significantly upregulated at 3dpi and 23dpi, indicating that *SoxE* and *Dmrt1* may be closely related to the development and maintenance of the testes. Unexpectedly, *foxl2* was significantly downregulated at 7dpi after *SoxE*-knockdown. Although RNAi provides a useful approach to study gene function in non-model species (Yang et al., 2017; Yu et al., 2014; Zhang et al., 2019), it is indeed a big challenge to ensure considerable knockdown efficiency among individuals especially in echinoderms with open-tube circulation. Hence, it is of great value to evaluate the functions of *SoxE* and *Dmrt1* in testes differentiation through gene knockout biotechnologies.

Apart from *Dmrt1*, which is highly expressed in the testes, *Dmrt2*, *3a*, *3b* and *5*, as well as *SoxE,* showed significantly higher expression in tube feet than in other adult tissues. Current studies have shown that the tube feet of the sea cucumber has a wide range of functions, including locomotion, feeding, chemoreception and light sensitivity respiration (Wang et al., 2014; Sun et al., 2013). According to previous reports, the expression pattern of *Dmrt2* (*2a*, *2b*) are species-specific, with expression primarily in both the ovaries and testes (Yamaguchi et al., 2006; Sheng et al., 2014; Su et al., 2015). In vertebrates, *Dmrt3* shows high expression in the testes and nervous system; accordingly, it has been speculated to be involved in the development of nerves and germ cells (Yamaguchi et al., 2006; Li et al., 2008; Dong et al., 2010). In contrast, large transcripts of *Dmrt3a* and *Dmrt3b* have been detected in coelomocytes, indicating that they might contribute to the regulation of immune functions. Surprisingly, the expression of *SoxE* was higher in the ovaries than the testes. In vertebrates, the transcription factor *Sox9* is highly expressed in developing male gonadal ridges and serves as the master regulator of Sertoli cell differentiation, playing a crucial role in sex determination (Kent et al., 1996; Jakob and Lovell-Badge., 2011). In invertebrates, *SoxE* has been detected in both the ovaries and testes and is mainly located in oocytes, spermatocytes and Sertoli cells (Santerre et al., 2014; Li et al., 2016; Yu et al., 2017). This indicates that *SoxE* may be involved in ovary development and differentiation as well as in the development of the testis in invertebrates.

## 5 Conclusion

In conclusion, this study identified and characterized the *SoxE* gene and *Dmrt* gene family in *A.japonicus* for the first time. A total of five *Dmrt* genes (*Dmrt1*, *2*, *3a*, *3b* and *5*) were identified and classified into four families based on phylogenetic analyses. Expression profile analyses demonstrated that the express patterns of *Dmrt* genes and the *SoxE* gene. Among them, *Dmrt1* was higher expressed in the testes with low expression in the ovaries. Knocking-down of *Dmrt1* led to the downregulation of *SoxE* expression and upregulation of the ovarian regulator *foxl2* in the testes. This indicates that *Dmrt1* may be a positive regulator of *SoxE* and may play a role in the development of the testes in the sea cucumber. In contrast, *SoxE* was more highly expressed in the ovaries than the testes, and the transcript level of *Dmrt1* was significantly decreased while *foxl2* expression was sharply increased in *SoxE* knockdown testes. This study provides a comprehensive overview of the *Dmrt* gene family and contributes to a better understanding of the role of the *Dmrt1* and *SoxE* genes in the sex determination and differentiation mechanisms of the sea cucumber.

## Data Availability

The original contributions presented in the study are included in the article/Supplementary Material, further inquiries can be directed to the corresponding author.

## Supplementary material

SUPPLEMENTARY FIGURE S1 Gene structure of *Dmrt* and *SoxE*. **(A)** Conserved DM domain diagram of *Dmrts.* **(B)** Schematic diagram of the gene structure of *Dmrts*. **(C)** Conserved HMG domain diagram of *SoxE*.

SUPPLEMENTARY FIGURE S2 Amino acid alignments of Dmrt1 with the homologs from other species. **(A)** Full length of amino acid alignments of Dmrt1. **(B)** DM domain alignments of Dmrt1. The identities relative to are exhibited at the end of each sequence. The boule frame indicates the conserved position of the DM domain.

SUPPLEMENTARY FIGURE S3 Amino acid alignments of Dmrt2 with the homologs from other species. **(A)** Full length of amino acid alignments of Dmrt2. **(B)** DM domain alignments of Dmrt2. The identities relative to are exhibited at the end of each sequence. The boule frame indicates the conserved position of the DM domain.

SUPPLEMENTARY FIGURE S4 Amino acid alignments of Dmrt3 with the homologs from other species. **(A)** Full length of amino acid alignments of Dmrt3. **(B)** DM domain alignments of Dmrt3. The identities relative to are exhibited at the end of each sequence. The boule frame indicates the conserved position of the DM domain.

SUPPLEMENTARY FIGURE S5 Amino acid alignments of Dmrt5 with the homologs from other species. **(A)** Full length of amino acid alignments of Dmrt5. **(B)** DM domain alignments of Dmrt5. The identities relative to are exhibited at the end of each sequence. The boule frame indicates the conserved position of the DM domain.

SUPPLEMENTARY FIGURE S6 Histological examination of testes after RNAi of *Dmrt1*. Bars=50 μm.

SUPPLEMENTARY FIGURE S7 Histological examination of testes after RNAi of *soxE*. Bars=50 μm.

SUPPLEMENTARY FIGURE S8 Gene synteny of Dmrt3 with the homologues from other species. Conserved gene blocks are represented in matching colors, and transcription orientations are indicated by arrows.
