# Supplementary figures and images for: Identification and functional analysis of Dmrt1 gene and the SoxE gene in the sexual development of sea cucumber, Apostichopus japonicus
Source: Front Genet. 2023 Jan 19;14:1097825. doi: 10.3389/fgene.2023.1097825 (PMC9894652; doi:10.3389/fgene.2023.1097825)

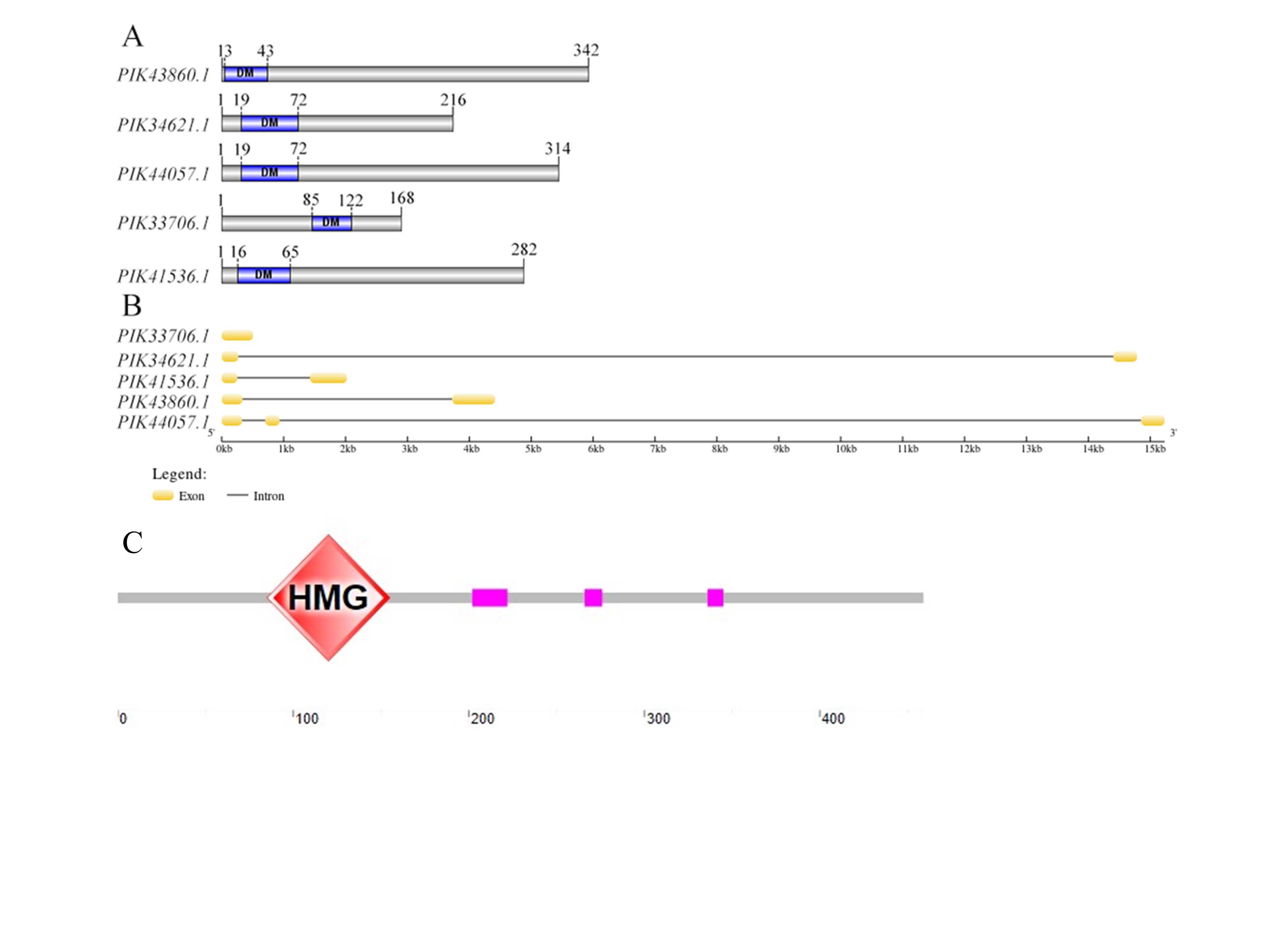

Supplement: Supplementary file 1 [file Image1.JPEG]

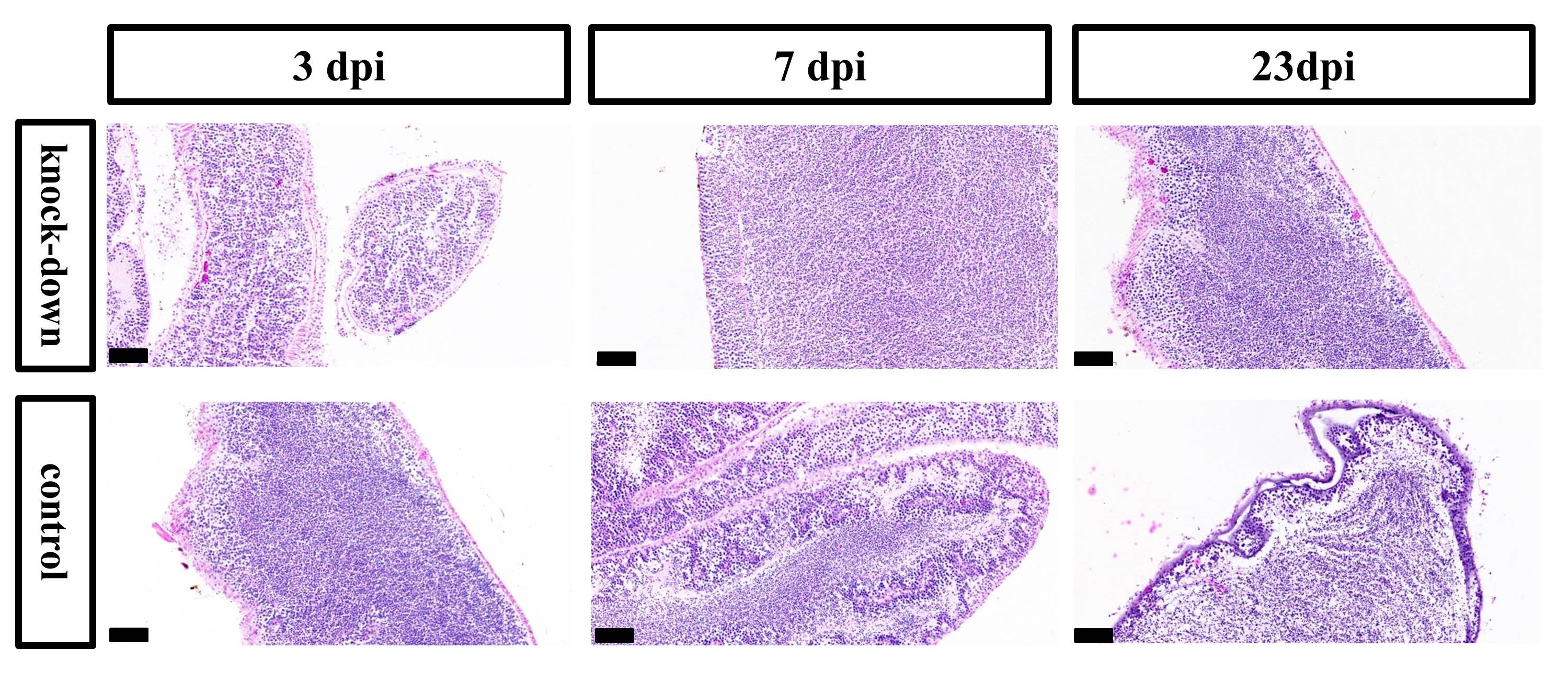

Supplement: Supplementary file 2 [file Image7.JPEG]

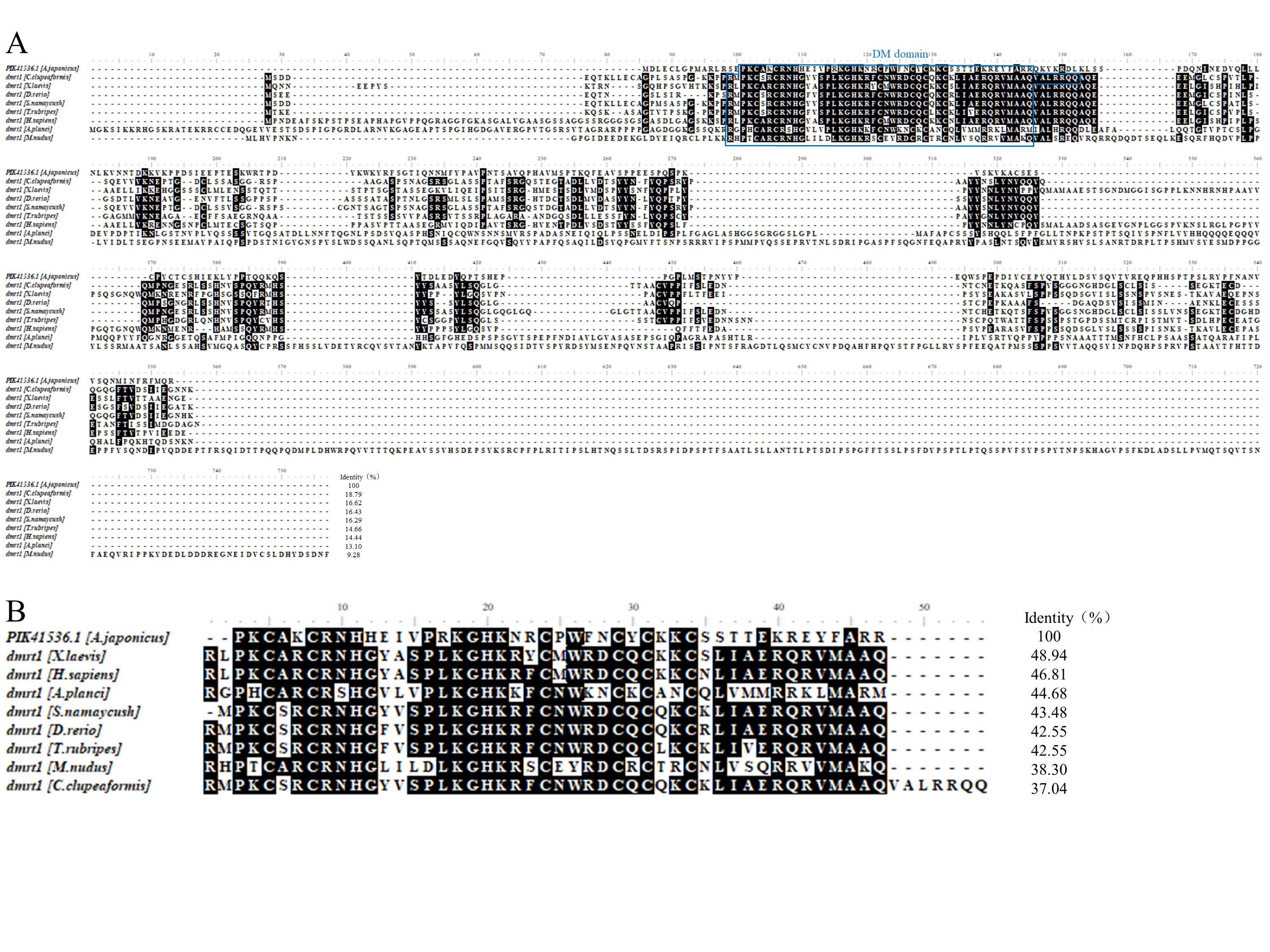

Supplement: Supplementary file 3 [file Image2.JPEG]

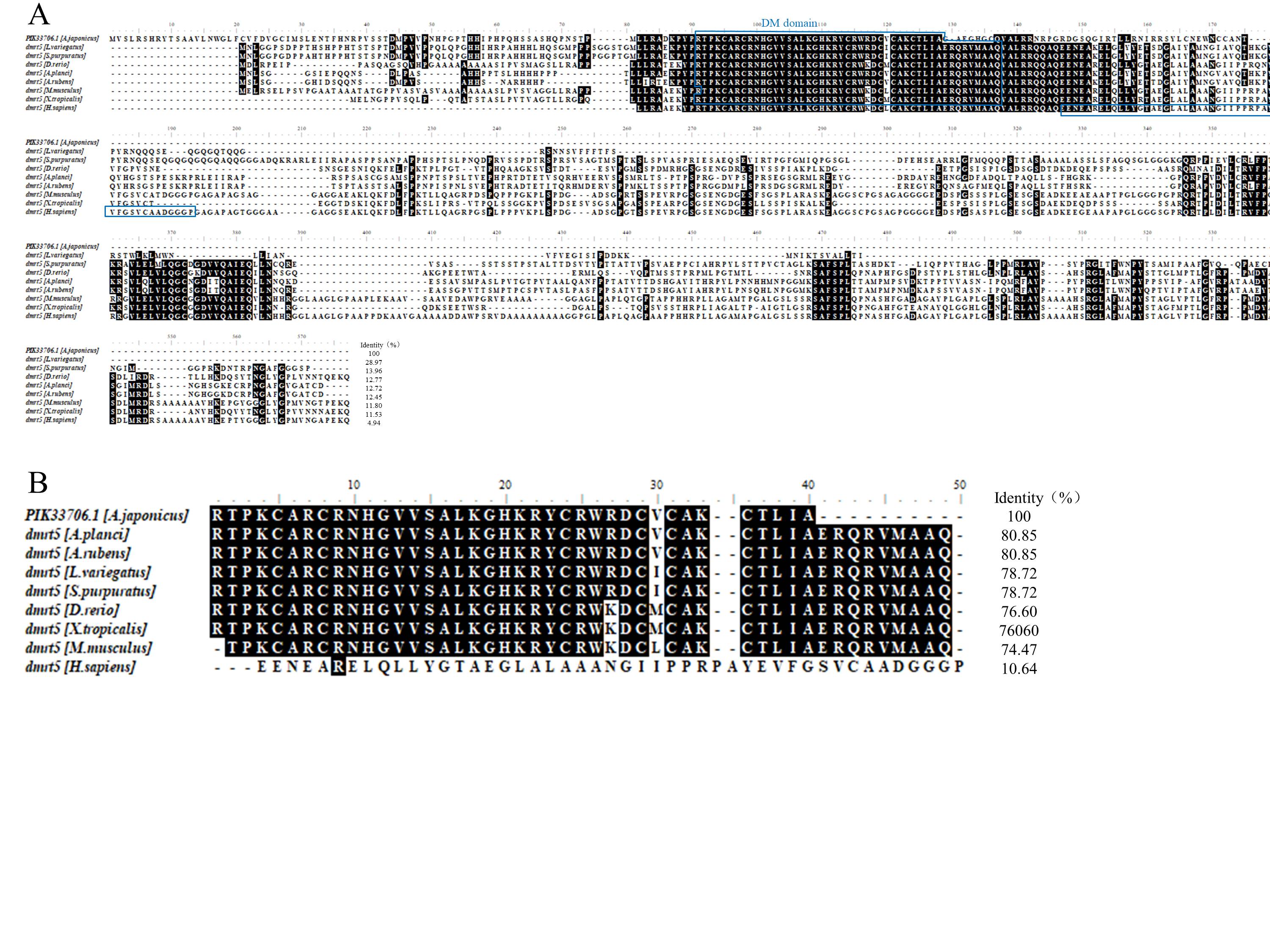

Supplement: Supplementary file 4 [file Image5.JPEG]

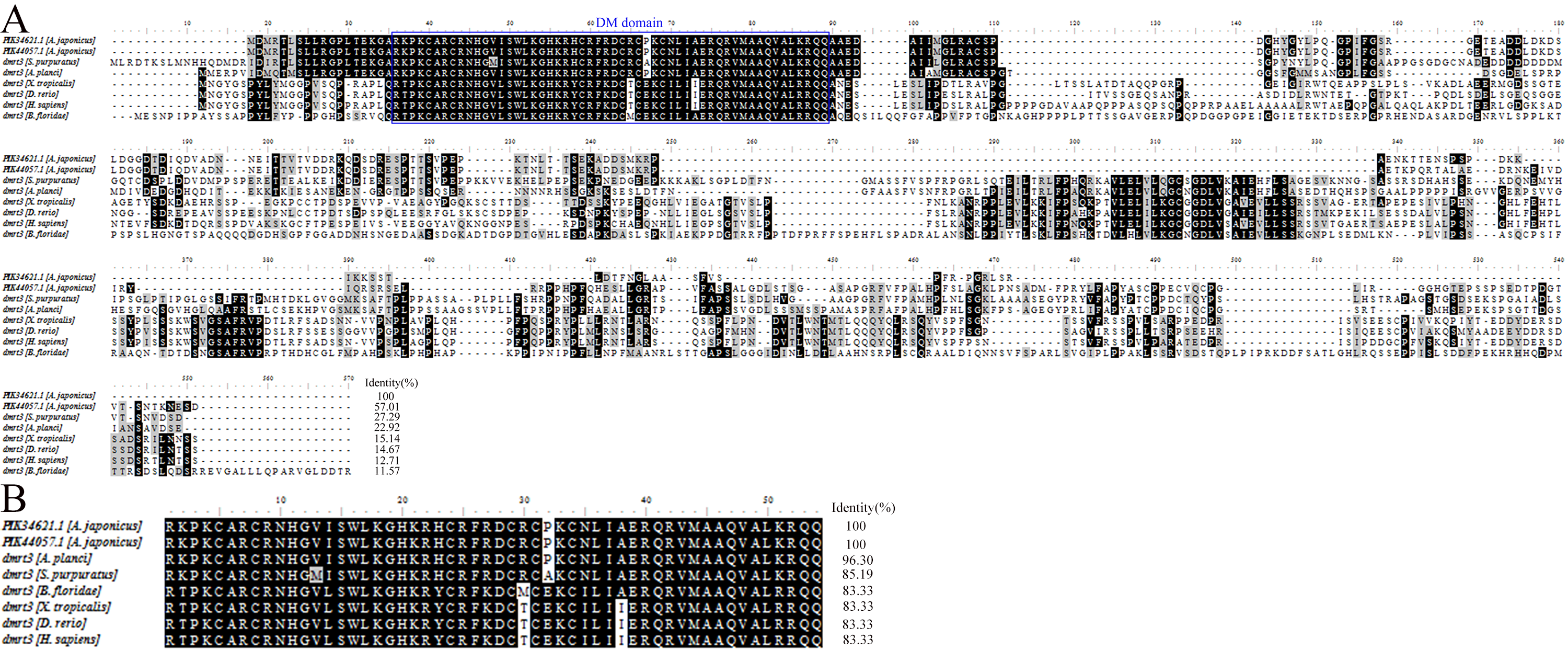

Supplement: Supplementary file 5 [file Image4.PNG]

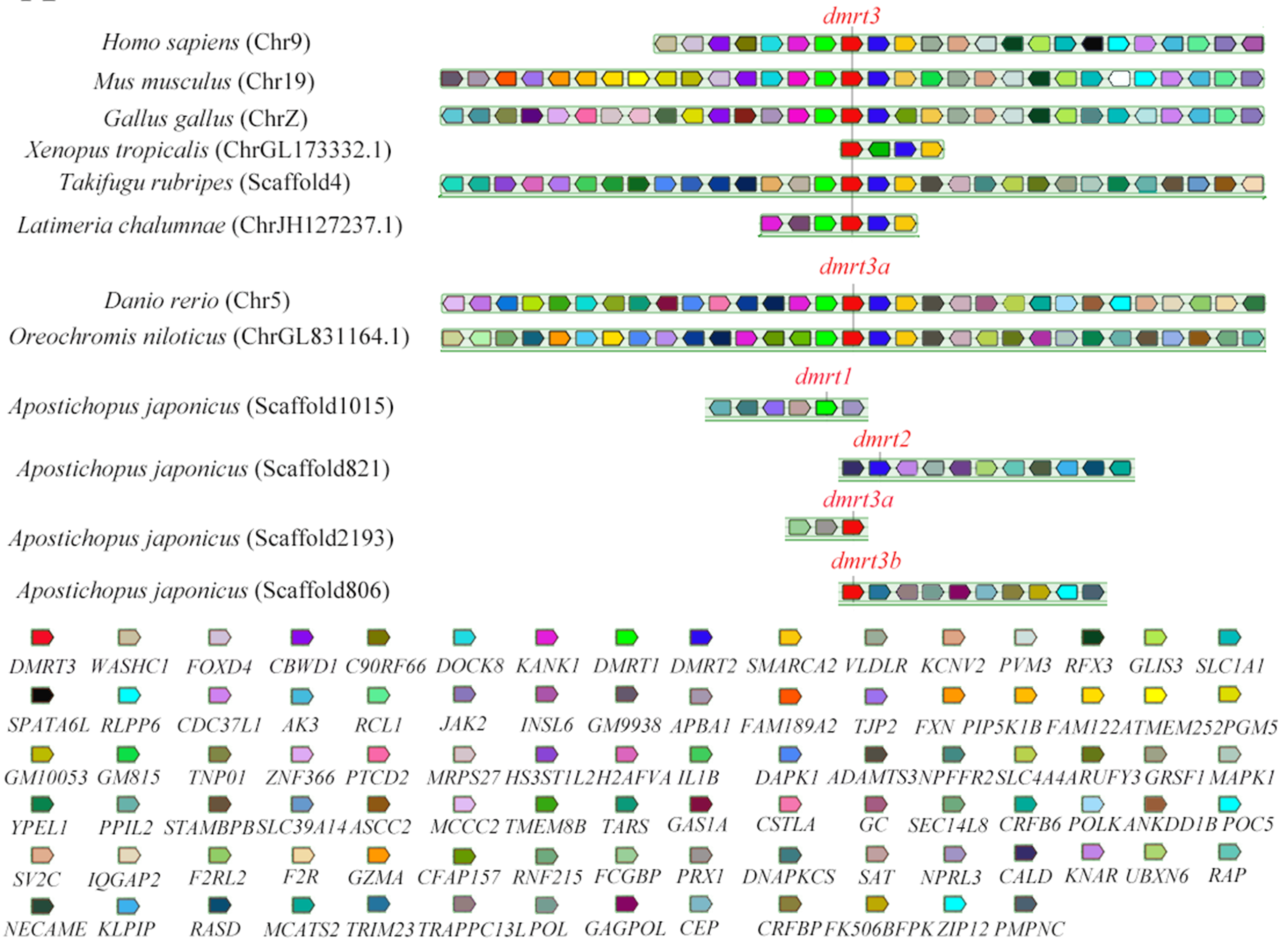

Supplement: Supplementary file 6 [file Image8.PNG]

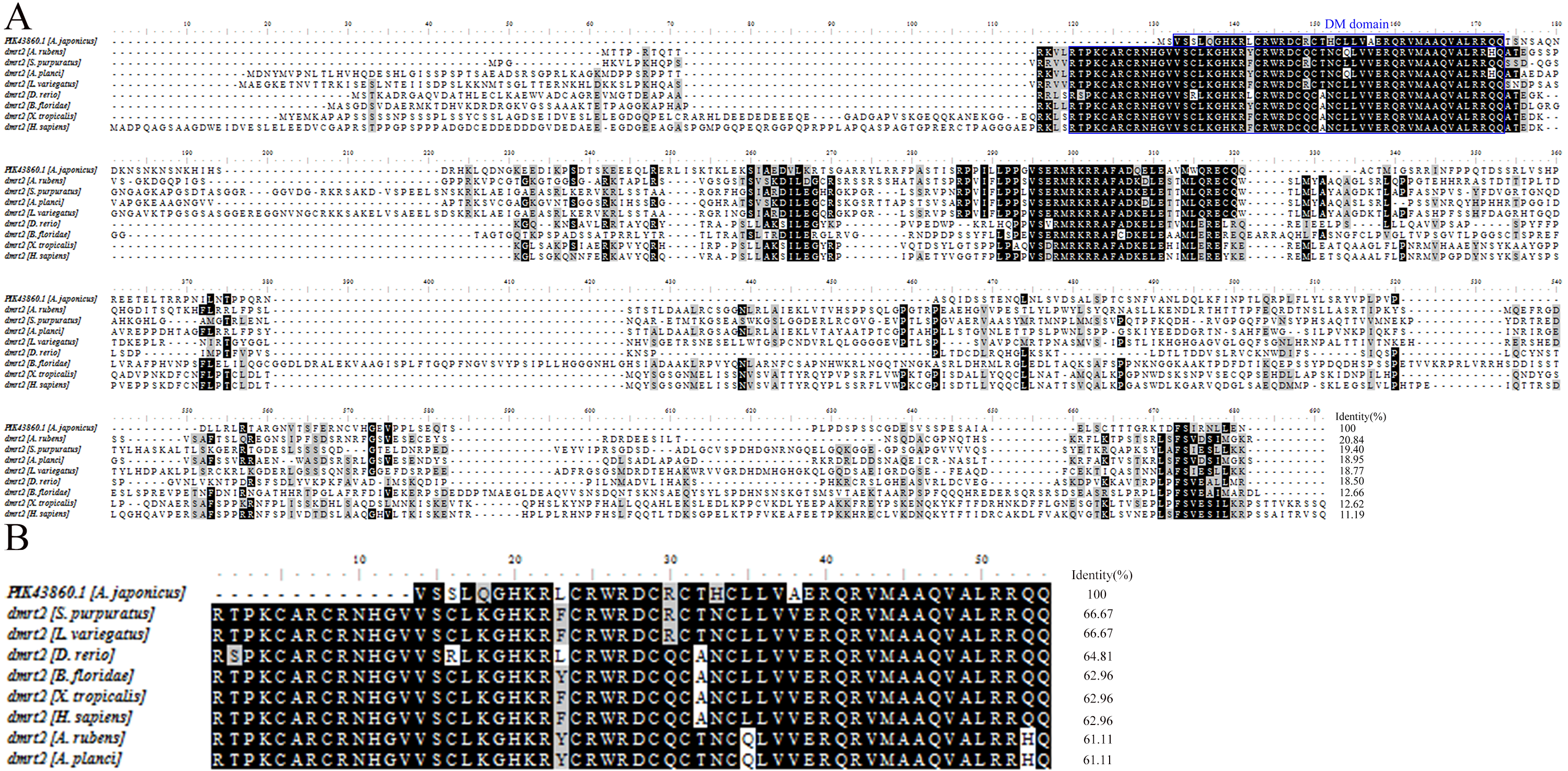

Supplement: Supplementary file 7 [file Image3.PNG]

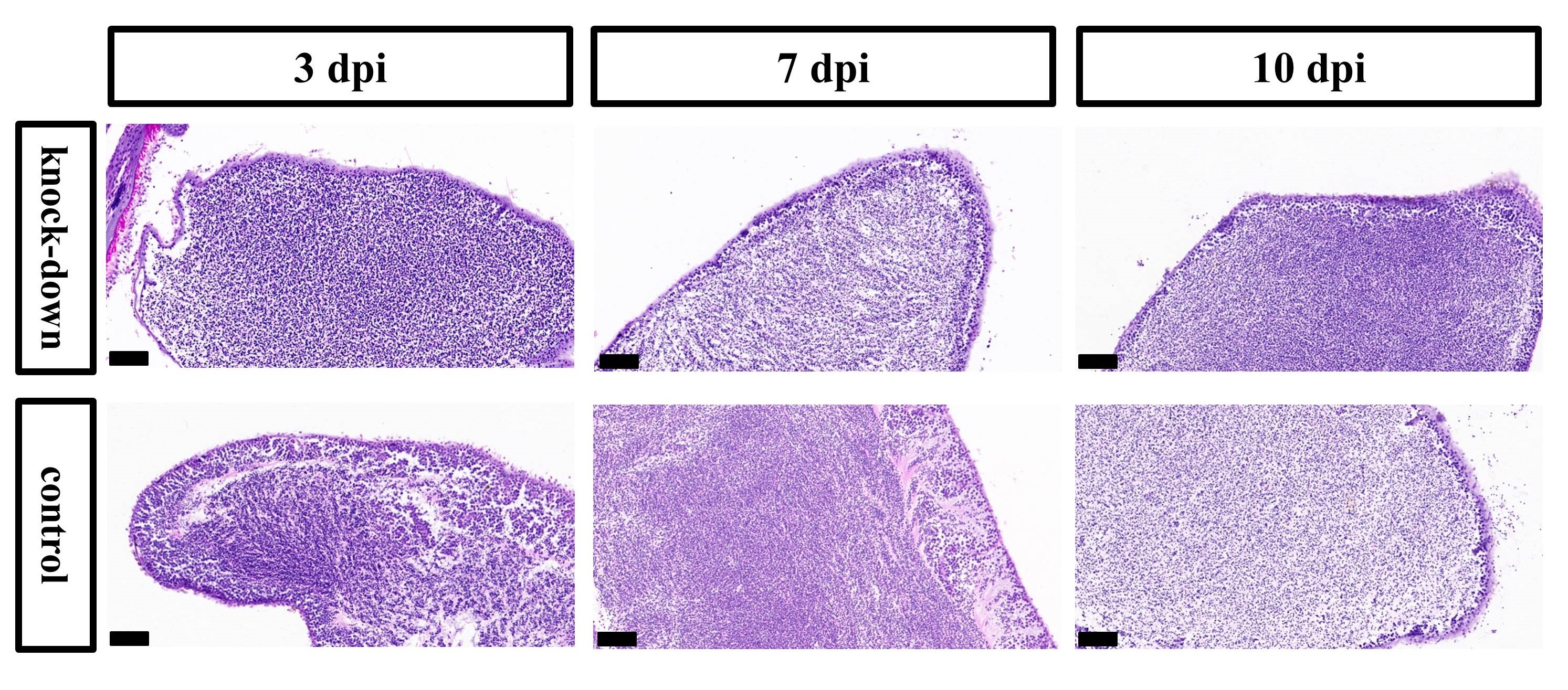

Supplement: Supplementary file 8 [file Image6.JPEG]
